# 2-(2-Nitro­phen­yl)acetohydrazide

**DOI:** 10.1107/S1600536812047381

**Published:** 2012-11-24

**Authors:** A. S. Praveen, Jerry P. Jasinski, Amanda C. Keeley, H. S. Yathirajan, B. Narayana

**Affiliations:** a Department of Studies in Chemistry, University of Mysore, Manasagangotri, Mysore 570 006, India; b Department of Chemistry, Keene State College, 229 Main Street, Keene, NH 03435-2001, USA; c Department of Studies in Chemistry, Mangalore University, Mangalagangotri 574 199, India

## Abstract

In the title compound, C_8_H_9_N_3_O_3_, the dihedral angle between the benzene ring and the acetohydrazide C—C(=O)—N—N plane [maximum deviation = 0.0471 (13) Å] is 87.62 (8)°. The nitro group is twisted by 19.3 (2)° with respect to the benzene ring. In the crystal, N—H⋯O hydrogen bonds link the mol­ecules into a double-column structure along the *b* axis.

## Related literature

For the chemistry of hydrazides, ses: Domiano *et al.* (1984 ▶). For the biological properties of hydrazides, see: Kalsi *et al.* (2006 ▶); Masunari & Tavares (2007 ▶); Singh *et al.* (1992 ▶). For related structures, see: Ahmad *et al.* (2012 ▶); Dutkiewicz *et al.* (2009 ▶); Liu & Gao (2012 ▶). For bond-length data, see: Allen *et al.* (1987 ▶).
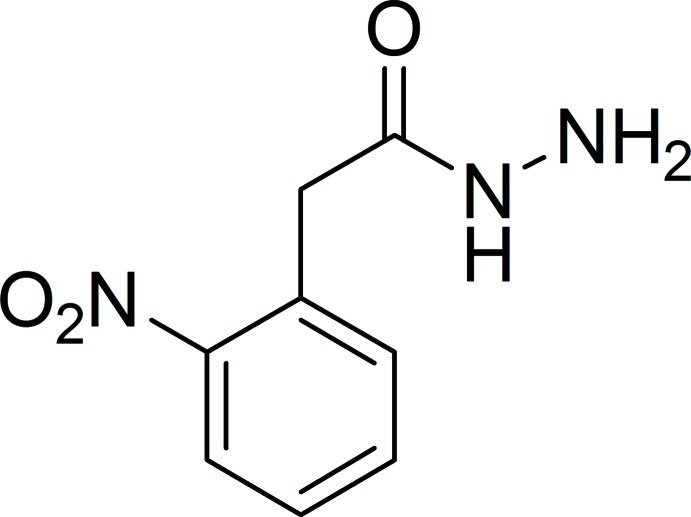


## Experimental

### 

#### Crystal data

C_8_H_9_N_3_O_3_
*M* *_r_* = 195.18
Monoclinic, 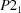

*a* = 6.6962 (5) Å
*b* = 4.9388 (4) Å
*c* = 13.3593 (12) Å
β = 92.361 (8)°
*V* = 441.43 (6) Å^3^
*Z* = 2
Cu *K*α radiation
μ = 0.98 mm^−1^
*T* = 173 K
0.36 × 0.28 × 0.08 mm

#### Data collection

Oxford Diffraction Xcalibur (Eos, Gemini) diffractometer
Absorption correction: multi-scan (*CrysAlis RED*; Oxford Diffraction, 2010 ▶) *T*_min_ = 0.667, *T*_max_ = 0.925
3829 measured reflections
1967 independent reflections
1824 reflections with *I* > 2σ(*I*)
*R*_int_ = 0.025

#### Refinement

*R*[*F* ^2^ > 2σ(*F* ^2^)] = 0.038
*wR*(*F* ^2^) = 0.096
*S* = 1.05
1967 reflections
136 parameters
4 restraints
H atoms treated by a mixture of independent and constrained refinement
Δρ_max_ = 0.19 e Å^−3^
Δρ_min_ = −0.17 e Å^−3^
Absolute structure: Flack (1983 ▶), 836 Friedel pairs
Flack parameter: 0.3 (3)

### 

Data collection: *CrysAlis PRO* (Oxford Diffraction, 2010 ▶); cell refinement: *CrysAlis PRO*; data reduction: *CrysAlis RED*; program(s) used to solve structure: *SHELXS97* (Sheldrick, 2008 ▶); program(s) used to refine structure: *SHELXL97* (Sheldrick, 2008 ▶); molecular graphics: *SHELXTL* (Sheldrick, 2008 ▶); software used to prepare material for publication: *SHELXTL*.

## Supplementary Material

Crystal structure: contains datablock(s) global, I. DOI: 10.1107/S1600536812047381/is5219sup1.cif

Structure factors: contains datablock(s) I. DOI: 10.1107/S1600536812047381/is5219Isup2.hkl

Supplementary material file. DOI: 10.1107/S1600536812047381/is5219Isup3.cml

Additional supplementary materials:  crystallographic information; 3D view; checkCIF report

## Figures and Tables

**Table 1:** Hydrogen-bond geometry (Å, °)

*D*—H⋯*A*	*D*—H	H⋯*A*	*D*⋯*A*	*D*—H⋯*A*
N1—H1*B*⋯O1^i^	0.90 (1)	2.21 (2)	3.0752 (19)	163 (2)
N2—H2⋯O1^ii^	0.85 (2)	2.03 (2)	2.8531 (18)	165 (2)

Symmetry codes: (i) 
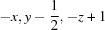
; (ii) 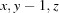.

**Fig. 1.**
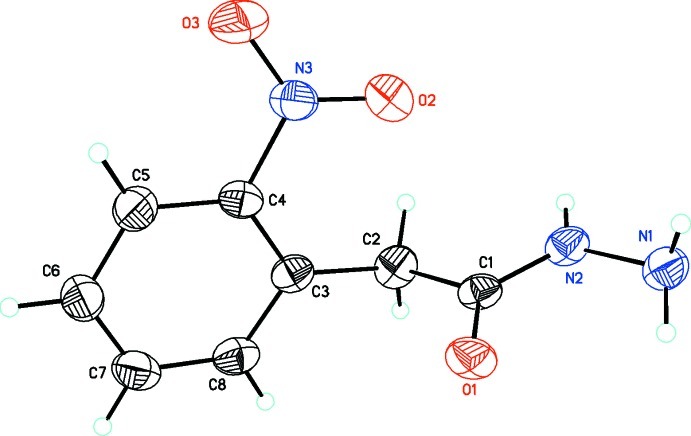
Molecular structure of the title compound showing the atom labeling scheme and 50% probability displacement ellipsoids.

**Fig. 2.**
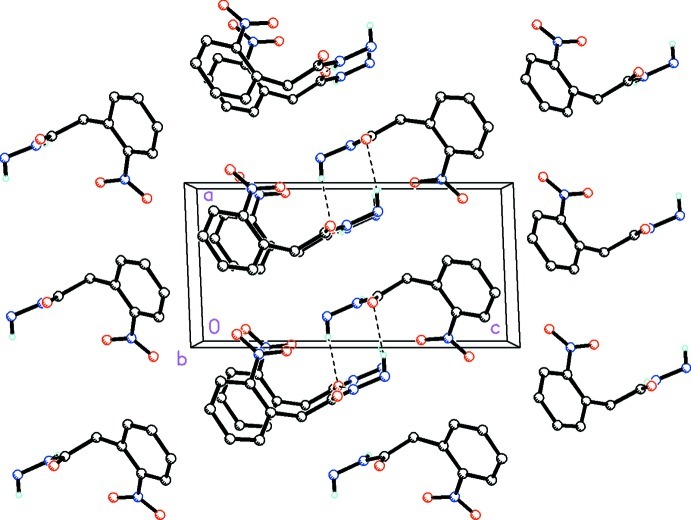
Packing diagram of the title compound viewed along the b axis. Dashed lines indicate N—H···O hydrogen bonds. H atoms not involved in the hydrogen bonds have been removed for clarity.

**Fig. 3.**
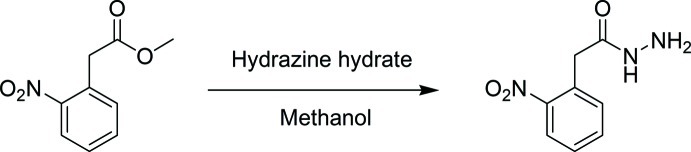
Synthesis of the title compound.

## supplementary crystallographic information

### Comment

The chemistry of hydrazides has been intensely investigated in recent years due to their excellent coordinating capability (Domiano *et al.*, 1984). Hydrazides and their condensation products have displayed diverse range of biological properties such as anti-helmintic (Kalsi *et al.*, 2006), anti-leprotic (Masunari & Tavares, 2007) and anti-depressant (Singh *et al.*, 1992). The crystal structures of some hydrazides, viz., 2-(4-bromophenyl)acetohydrazide (Ahmad *et al.*, 2012), 2-(4-chlorophenoxy)acetohydrazide (Dutkiewicz *et al.*, 2009) and 2-(4-methoxyphenoxy)acetohydrazide (Liu & Gao, 2012) have been reported. In view of the importance of hydrazides, the crystal structure of title compound (I) is reported.

In the title compound, the dihedral angle between the benzene ring and acetohydrazide C2/C1/O1/N2/N1 plane is 87.62 (8)° (Fig. 1). The nitro group is twisted by 19.3 (2)° with the benzene ring. Bond lengths are in normal ranges (Allen *et al.*, 1987). In the crystal, N—H···O hydrogen bonds (Table 1) link the molecules into a double-column structure along the *b* axis (Fig. 2).

### Experimental

To a solution of methyl 2-(2-nitrophenyl)acetate (2 g, 10.14 mmol) in methanol (20 mL), hydrazine hydrate (2 mL) was added and the reaction mixture was stirred at room temperature for 8 hours (Fig. 3). After the completion of the reaction, methanol was removed under vacuum, water was added, precipitated solid was filtered and dried. The single crystal was grown from mixture methanol: water (2:1) by slow evaporation method and yield of the compound was 95%. (m.p.: 422-424 K).

### Refinement

Atoms H1A, H1B and H2 were refined with a bond-length restraint N—H = 0.86 (2) Å. All remaining H atoms were placed in their calculated positions and then refined using the riding model with C—H lengths of 0.93 Å (CH) and 0.97 Å (CH_2_). Isotropic displacement parameters were set to 1.2 times *U*_eq_ of the parent atom. The Flack parameter 0.3 (3) and the Hooft y parameter of 0.45 (18) imply that the crystal used was an inversion twin.

### Crystal data

**Table:**

C_8_H_9_N_3_O_3_	*F*(000) = 204
*M**_r_* = 195.18	*D*_x_ = 1.468 Mg m^−^^3^
Monoclinic, *P*2_1_	Cu *K*α radiation, λ = 1.54184 Å
Hall symbol: P 2yb	Cell parameters from 1694 reflections
*a* = 6.6962 (5) Å	θ = 3.3–32.5°
*b* = 4.9388 (4) Å	µ = 0.98 mm^−^^1^
*c* = 13.3593 (12) Å	*T* = 173 K
β = 92.361 (8)°	Chunk, colorless
*V* = 441.43 (6) Å^3^	0.36 × 0.28 × 0.08 mm
*Z* = 2	

### Data collection

**Table:**

Oxford Diffraction Xcalibur (Eos, Gemini) diffractometer	1967 independent reflections
Radiation source: Enhance (Mo) X-ray Source	1824 reflections with *I* > 2σ(*I*)
Graphite monochromator	*R*_int_ = 0.025
Detector resolution: 16.0416 pixels mm^-1^	θ_max_ = 89.1°, θ_min_ = 7.3°
ω scans	*h* = −8→8
Absorption correction: multi-scan (*CrysAlis RED*; Oxford Diffraction, 2010)	*k* = −6→6
*T*_min_ = 0.667, *T*_max_ = 0.925	*l* = −17→17
3829 measured reflections	

### Refinement

**Table:**

Refinement on *F*^2^	Secondary atom site location: difference Fourier map
Least-squares matrix: full	Hydrogen site location: inferred from neighbouring sites
*R*[*F*^2^ > 2σ(*F*^2^)] = 0.038	H atoms treated by a mixture of independent and constrained refinement
*wR*(*F*^2^) = 0.096	*w* = 1/[σ^2^(*F*_o_^2^) + (0.0517*P*)^2^ + 0.016*P*] where *P* = (*F*_o_^2^ + 2*F*_c_^2^)/3
*S* = 1.05	(Δ/σ)_max_ < 0.001
1967 reflections	Δρ_max_ = 0.19 e Å^−^^3^
136 parameters	Δρ_min_ = −0.17 e Å^−^^3^
4 restraints	Absolute structure: Flack (1983), 836 Friedel pairs
Primary atom site location: structure-invariant direct methods	Flack parameter: 0.3 (3)

### Special details

**Table:**

Geometry. All esds (except the esd in the dihedral angle between two l.s. planes) are estimated using the full covariance matrix. The cell esds are taken into account individually in the estimation of esds in distances, angles and torsion angles; correlations between esds in cell parameters are only used when they are defined by crystal symmetry. An approximate (isotropic) treatment of cell esds is used for estimating esds involving l.s. planes.
Refinement. Refinement of *F*^2^ against ALL reflections. The weighted *R*-factor *wR* and goodness of fit *S* are based on *F*^2^, conventional *R*-factors *R* are based on *F*, with *F* set to zero for negative *F*^2^. The threshold expression of *F*^2^ > σ(*F*^2^) is used only for calculating *R*-factors(gt) *etc*. and is not relevant to the choice of reflections for refinement. *R*-factors based on *F*^2^ are statistically about twice as large as those based on *F*, and *R*- factors based on ALL data will be even larger.

### Fractional atomic coordinates and isotropic or equivalent isotropic displacement parameters (Å^2^)

**Table:**

	*x*	*y*	*z*	*U*_iso_*/*U*_eq_	
O1	0.27090 (17)	0.8745 (2)	0.55928 (9)	0.0360 (3)	
O2	0.0246 (2)	0.4300 (4)	0.71073 (10)	0.0632 (5)	
O3	−0.0790 (2)	0.4790 (4)	0.85895 (11)	0.0576 (4)	
N1	0.1751 (2)	0.4904 (3)	0.41437 (10)	0.0360 (3)	
H1A	0.202 (3)	0.660 (4)	0.4013 (15)	0.043*	
H1B	0.042 (2)	0.494 (5)	0.4186 (13)	0.043*	
N2	0.2580 (2)	0.4374 (3)	0.51138 (10)	0.0318 (3)	
H2	0.286 (3)	0.275 (4)	0.5269 (13)	0.038*	
N3	0.0398 (2)	0.5265 (3)	0.79401 (10)	0.0358 (4)	
C1	0.3052 (2)	0.6316 (3)	0.57676 (12)	0.0274 (3)	
C2	0.4158 (2)	0.5384 (4)	0.67189 (12)	0.0338 (4)	
H2A	0.3753	0.3547	0.6868	0.041*	
H2B	0.5580	0.5363	0.6609	0.041*	
C3	0.3777 (2)	0.7162 (3)	0.76100 (11)	0.0290 (3)	
C4	0.2066 (2)	0.7141 (3)	0.81839 (11)	0.0293 (3)	
C5	0.1834 (2)	0.8801 (4)	0.90089 (12)	0.0358 (4)	
H5	0.0682	0.8696	0.9372	0.043*	
C6	0.3327 (3)	1.0607 (4)	0.92857 (13)	0.0391 (4)	
H6	0.3182	1.1750	0.9831	0.047*	
C7	0.5050 (3)	1.0703 (4)	0.87408 (13)	0.0392 (4)	
H7	0.6064	1.1916	0.8922	0.047*	
C8	0.5260 (2)	0.9000 (4)	0.79295 (12)	0.0338 (4)	
H8	0.6435	0.9080	0.7583	0.041*	

### Atomic displacement parameters (Å^2^)

**Table:**

	*U*^11^	*U*^22^	*U*^33^	*U*^12^	*U*^13^	*U*^23^
O1	0.0441 (6)	0.0188 (6)	0.0449 (7)	0.0021 (5)	−0.0010 (5)	0.0046 (5)
O2	0.0697 (9)	0.0736 (12)	0.0466 (8)	−0.0375 (9)	0.0085 (6)	−0.0133 (8)
O3	0.0440 (7)	0.0653 (11)	0.0651 (9)	−0.0193 (7)	0.0216 (6)	−0.0069 (8)
N1	0.0431 (7)	0.0306 (8)	0.0346 (7)	−0.0004 (7)	0.0062 (6)	0.0013 (6)
N2	0.0423 (7)	0.0197 (7)	0.0337 (7)	0.0046 (6)	0.0070 (5)	0.0036 (6)
N3	0.0339 (7)	0.0332 (9)	0.0405 (8)	−0.0061 (6)	0.0037 (6)	0.0013 (6)
C1	0.0296 (7)	0.0197 (8)	0.0336 (8)	0.0031 (6)	0.0087 (6)	0.0028 (6)
C2	0.0387 (8)	0.0261 (9)	0.0369 (9)	0.0096 (7)	0.0048 (6)	0.0034 (7)
C3	0.0314 (7)	0.0254 (8)	0.0303 (7)	0.0034 (6)	0.0006 (6)	0.0079 (7)
C4	0.0288 (7)	0.0242 (8)	0.0347 (8)	−0.0011 (6)	0.0007 (6)	0.0043 (7)
C5	0.0385 (8)	0.0353 (10)	0.0338 (8)	−0.0005 (8)	0.0048 (6)	0.0011 (7)
C6	0.0509 (10)	0.0333 (10)	0.0330 (9)	−0.0026 (8)	−0.0003 (7)	−0.0012 (7)
C7	0.0432 (9)	0.0332 (10)	0.0404 (9)	−0.0089 (8)	−0.0076 (7)	0.0077 (8)
C8	0.0300 (7)	0.0351 (10)	0.0362 (8)	−0.0023 (7)	0.0003 (6)	0.0095 (7)

### Geometric parameters (Å, º)

**Table:**

O1—C1	1.242 (2)	C2—H2B	0.9700
O2—N3	1.2107 (19)	C3—C8	1.399 (2)
O3—N3	1.2236 (18)	C3—C4	1.405 (2)
N1—N2	1.413 (2)	C4—C5	1.387 (2)
N1—H1A	0.877 (16)	C5—C6	1.379 (3)
N1—H1B	0.898 (14)	C5—H5	0.9300
N2—C1	1.327 (2)	C6—C7	1.390 (3)
N2—H2	0.845 (16)	C6—H6	0.9300
N3—C4	1.477 (2)	C7—C8	1.384 (3)
C1—C2	1.516 (2)	C7—H7	0.9300
C2—C3	1.509 (2)	C8—H8	0.9300
C2—H2A	0.9700		
			
N2—N1—H1A	106.5 (14)	C8—C3—C4	115.04 (15)
N2—N1—H1B	107.5 (12)	C8—C3—C2	118.50 (14)
H1A—N1—H1B	102 (2)	C4—C3—C2	126.45 (15)
C1—N2—N1	122.93 (15)	C5—C4—C3	123.33 (15)
C1—N2—H2	118.6 (13)	C5—C4—N3	115.94 (13)
N1—N2—H2	118.3 (13)	C3—C4—N3	120.72 (14)
O2—N3—O3	122.93 (16)	C6—C5—C4	119.47 (15)
O2—N3—C4	118.92 (13)	C6—C5—H5	120.3
O3—N3—C4	118.13 (14)	C4—C5—H5	120.3
O1—C1—N2	122.50 (16)	C5—C6—C7	119.32 (16)
O1—C1—C2	122.07 (16)	C5—C6—H6	120.3
N2—C1—C2	115.32 (15)	C7—C6—H6	120.3
C3—C2—C1	113.10 (14)	C8—C7—C6	120.18 (17)
C3—C2—H2A	109.0	C8—C7—H7	119.9
C1—C2—H2A	109.0	C6—C7—H7	119.9
C3—C2—H2B	109.0	C7—C8—C3	122.65 (15)
C1—C2—H2B	109.0	C7—C8—H8	118.7
H2A—C2—H2B	107.8	C3—C8—H8	118.7
			
N1—N2—C1—O1	3.6 (2)	O3—N3—C4—C5	−18.1 (2)
N1—N2—C1—C2	−172.72 (13)	O2—N3—C4—C3	−20.3 (2)
O1—C1—C2—C3	32.4 (2)	O3—N3—C4—C3	160.87 (17)
N2—C1—C2—C3	−151.21 (14)	C3—C4—C5—C6	1.0 (2)
C1—C2—C3—C8	−103.44 (17)	N3—C4—C5—C6	179.95 (15)
C1—C2—C3—C4	77.9 (2)	C4—C5—C6—C7	−0.9 (3)
C8—C3—C4—C5	0.0 (2)	C5—C6—C7—C8	0.0 (3)
C2—C3—C4—C5	178.63 (16)	C6—C7—C8—C3	1.0 (3)
C8—C3—C4—N3	−178.96 (14)	C4—C3—C8—C7	−1.0 (2)
C2—C3—C4—N3	−0.3 (2)	C2—C3—C8—C7	−179.73 (16)
O2—N3—C4—C5	160.74 (17)		

### Hydrogen-bond geometry (Å, º)

**Table:**

*D*—H···*A*	*D*—H	H···*A*	*D*···*A*	*D*—H···*A*
N1—H1*B*···O1^i^	0.90 (1)	2.21 (2)	3.0752 (19)	163 (2)
N2—H2···O1^ii^	0.85 (2)	2.03 (2)	2.8531 (18)	165 (2)

Symmetry codes: (i) −*x*, *y*−1/2, −*z*+1; (ii) *x*, *y*−1, *z*.
